# Supplementary material for: Splice-Junction-Based Mapping of Alternative Isoforms in the Human Proteome
Source: Cell Rep. Author manuscript; Available in PMC 2020 Jan 15. (PMC6961840; doi:10.1016/j.celrep.2019.11.026)
Supplement: 3 [file NIHMS1546469-supplement-3.zip › DF2/PXD006675/CardiacFibroblast-130-Q9UH99-SSLEELHGDANWGYSDVDQQSSSSR.pdf]

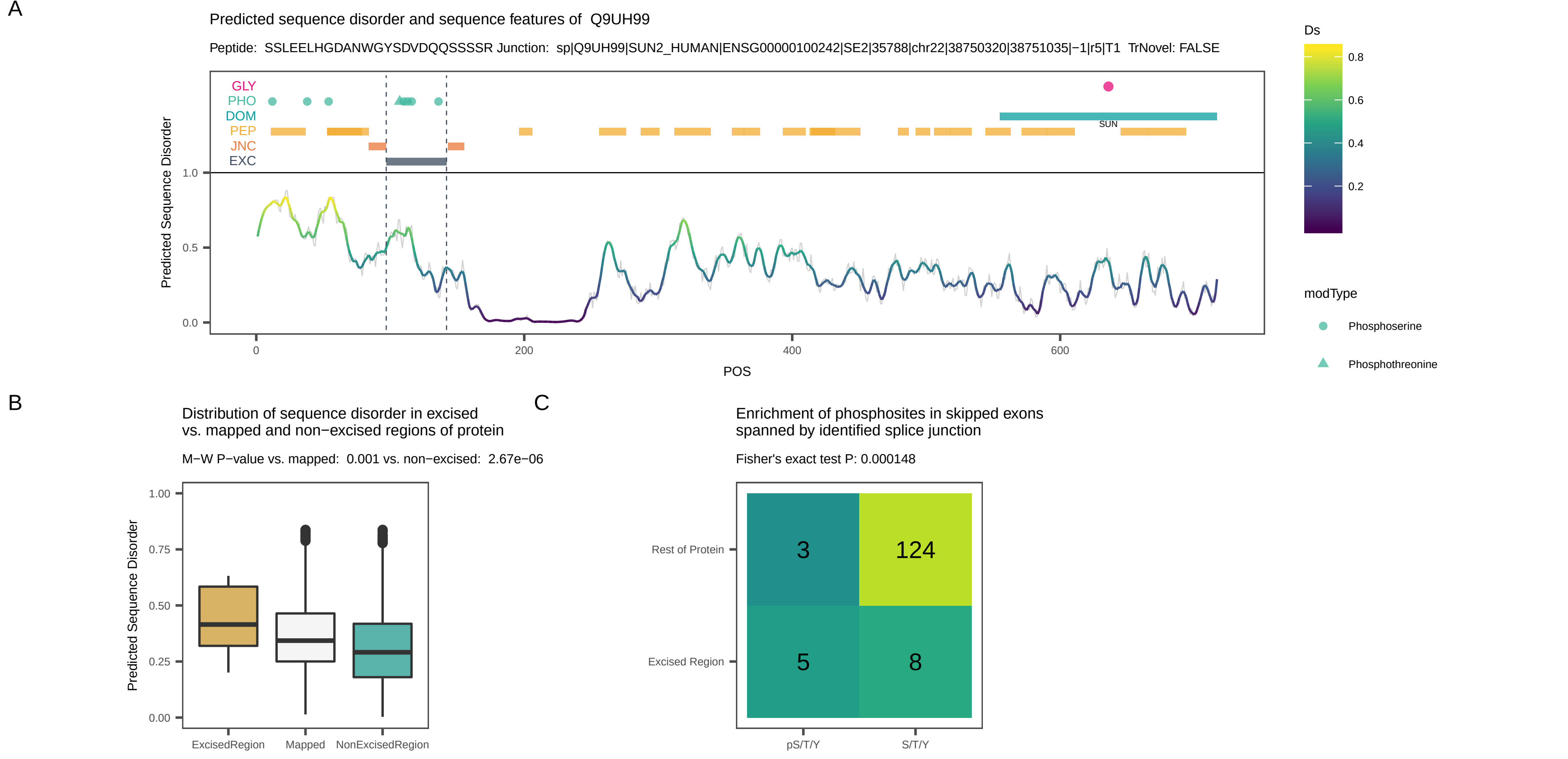
A
Predicted sequence disorder and sequence features of Q9UH99
Peptide: SSLEELHGDANWGYSDVDQQSSSSR Junction: sp|Q9UH99|SUN2_HUMAN|ENSG00000100242|SE2|35788|chr22|38750320|38751035|−1|r5|T1 TrNovel: FALSE
GLY
PHO
DOM
PEP
JNC
EXC
SUN
Predicted Sequence Disorder
1.0
0.5
0.0
0
200
400
600
POS
Ds
0.8
0.6
0.4
0.2
modType
Phosphoserine
Phosphothreonine
B
Distribution of sequence disorder in excised vs. mapped and non−excised regions of protein
M−W P−value vs. mapped: 0.001 vs. non−excised: 2.67e−06
Predicted Sequence Disorder
1.00
0.75
0.50
0.25
0.00
ExcisedRegion
Mapped
NonExcisedRegion
C
Enrichment of phosphosites in skipped exons spanned by identified splice junction
Fisher's exact test P: 0.000148
Rest of Protein
Excised Region
3
124
5
8
pS/T/Y
S/T/Y
